# Characterization of Multi-antibiotic-resistant *Escherichia coli* Isolated from Beef Cattle in Japan

**DOI:** 10.1264/jsme2.ME13173

**Published:** 2014-04-30

**Authors:** Shiori Yamamoto, Motoki Nakano, Wataru Kitagawa, Michiko Tanaka, Teruo Sone, Katsuya Hirai, Kozo Asano

**Affiliations:** 1 Applied Microbiology, Graduate School of Agriculture, Hokkaido University, Kita-ku, Sapporo, Hokkaido 060–8589, Japan; 2 Bioproduction Research Institute, National Institute of Advanced Industrial Science and Technology (AIST), Toyohira-ku, Sapporo 062–8517, Japan; 3 Department of Nutrition, School of Nursing and Nutrition, Tenshi College, Higashi-ku, Sapporo, Hokkaido 065–0013, Japan

**Keywords:** multiple-antibiotic resistance, plasmids, *Escherichia coli*, resistance gene

## Abstract

The emergence of multiple-antibiotic-resistance bacteria is increasing, which is a particular concern on livestock farms. We previously isolated 1,347 antimicrobial-resistant (AMR) *Escherichia coli* strains from the feces of beef cattle on 14 Japanese farms. In the present study, the genetic backgrounds and phylogenetic relationships of 45 AMR isolates were characterized by the chromosome phylotype, AMR phenotype, AMR genotype, and plasmid type. These isolates were classified into five chromosome phylotypes, which were closely linked to the farms from which they were isolated, suggesting that each farm had its own *E. coli* phylotype. AMR phenotype and plasmid type analyses yielded 8 and 14 types, all of which were associated with the chromosomal phylotype and, thus, to the original farms. AMR genotype analysis revealed more variety, with 16 types, indicating both inter- and intra-farm diversity. Different phylotype isolates from the same farm shared highly similar plasmid types, which indicated that plasmids with AMR genes could be transferred between phylotypes, thereby generating multi-antibiotic-resistant microorganisms. This ecological study demonstrated that the chromosome phylotype was strongly correlated with the farm from which they were isolated, while the AMR phenotype, genotype, and plasmid type were generally correlated with the chromosome phylotype and farm source.

Antimicrobial resistance (AMR) is a serious concern in human medicine and public health, and the emergence of multiple-antibiotic-resistant bacteria constitutes a global problem. The most commonly encountered AMR bacteria are *Escherichia coli*, *Enterococcus* spp., *Salmonella* spp., and *Staphylococcus* spp. (1). Intestinal *E. coli* provide a reservoir for transmissible AMR factors (41) and have been used as an indicator for AMR strains (1). Commensal *E. coli* have a broad host-range in warm-blooded animals including humans (2), and can transfer resistance genes from commensals to pathogenic strains. AMR strains in livestock may transfer their AMR genes to humans via food animals and environmental contact.

Bacterial AMR profiles, such as the number of antimicrobial agents, were previously shown to be strengthened when antimicrobial agents were used under selective pressure (8, 36). Therefore, using antimicrobial agents, and their types, doses, and locations, are known to influence the variety and distribution of AMR strains and resistance genes (20). Antimicrobial agents are used in human medicine, livestock farms, and aquaculture. The total consumption of veterinary antimicrobial agents in Japan increased from 970 tons per year in 2000 to 1,060 tons in 2001, and subsequently decreased to 870 tons in 2005 (http://www.maff.go.jp/nval/tyosa_kenkyu/taiseiki/index.html). The use of antibiotics in feed averaged 171 tons per year from 2000 to 2005 (http://www.maff.go.jp/nval/tyosa_kenkyu/taiseiki/index.html). However, the amount of veterinary antimicrobial agents (therapeutic and feed additive) sold per food-producing animal weight (pig, broiler, and cattle) in Japan increased from 132 mg kg^−1^ to 153 mg kg^−1^ from 2005 to 2010 (25). The use of antibiotics in food animals is increasing, as is the frequency of AMR strains on livestock farms (25, 43, http://www.maff.go.jp/nval/tyosa_kenkyu/taiseiki/index.html).

In 2009, we obtained 3,147 *E. coli* isolates from the feces of beef cattle on 14 farms in three Japanese regions (Hokkaido, Chubu, and Kyushu) (43) and assessed these isolates for antibiotic resistance. We found that 44.4% (1,347 isolates) of the isolates were AMR, which represented a higher frequency than previously reported (13, 24, 40). For example, a study conducted in 1994 cited a frequency of 30.6% AMR *E. coli* isolates from cattle (24). Thus, the frequency of AMR strains is increasing. Our preliminary study also demonstrated that AMR properties were farm-specific, possibly due to varying AMR gene distributions. However, AMR profiles and their associated AMR gene have yet to be investigated by region.

AMR properties are conferred by resistance genes encoding (i) drug-inactivating enzymes, (ii) reduced membrane permeability, and (iii) antibiotic efflux pumps, or are caused by mutations in antibiotic target sites (28). AMR genes located on mobile elements such as plasmids, transposons, and integrons can be exchanged between strains. Plasmids are major genetic vectors and each has its own host range, transmissibility, and stability characteristics (17). These characteristics are responsible for capturing foreign DNAs such as AMR genes, and their complex mosaic structure allows them to confer multiple-antibiotic resistance to the host microorganisms (23). One of the most important plasmid characteristics is incompatibility, which allows for the coexistence of different plasmid types, each carrying different AMR genes, leading to multiple-antibiotic resistance. At least 18 incompatibility types have been identified to date, some of which have been associated with multiple-antibiotic resistance in *E. coli* (5, 15, 18). For example, IncA/C-type plasmids have been shown to contain multiple-antibiotic resistance mobile elements such as *bla*_CMY-2_ for beta-lactam resistance, *aadA* for streptomycin resistance, and *sul* for sulfa resistance (5, 15, 18). The relationships between AMR profiles and AMR genes and between plasmid phylotype, replicon type, and AMR genes have been investigated previously (33, 35); however, a combined analysis of these profiles with the chromosome phylotype has not yet been conducted. There have also been no comprehensive studies on the chromosome phylotype, AMR profile, AMR genes, and plasmid incompatibility types in multidrug-resistant strains.

In the present study, we examined 45 multiple-antibiotic resistant *E. coli* isolates from five different beef cattle farms in Japan. These isolates were resistant to nine or more antimicrobial agents and were selected from the 3,147 isolates obtained in our previous study (43). To understand the genetic backgrounds and phylogenetic relationships of AMR in these isolates, we analyzed their chromosome phylotype, AMR phenotype, AMR genotype, and plasmid incompatibility type. We also elucidated the relationship between these characteristics and the origins of each isolate. This is the first study to examine the ecological relationships and genetic characteristics of multiple-antibiotic resistant *E. coli* on beef cattle farms.

## Materials and Methods

### Chromosome phylotype analysis

Phylotypes were determined by restriction fragment length polymorphism (RFLP) analysis with *Xba*I (Takara Bio, Otsu, Japan) digestion of whole genomic DNA, followed by PFGE (see below for PFGE conditions) (38). RFLP profiles were used to calculate the relative distance and dendrograms were constructed by the Ward method with Mulcel (OMS, Tokyo, Japan) running in Microsoft Office Excel.

### Bacteria and AMR phenotype analysis

In our previous study, 3,147 *E. coli* were isolated from beef cattle fecal samples in Hokkaido, Chubu, and Kyushu, Japan (43). In the present study, 45 strains of *E. coli* resistant to more than nine agents were selected (Table 1). These isolates were named by farm and cattle number; for example, “SA1-4-1” referred to cow 1-4-1 on the SA farm. Samples were collected from five farms: two in Hokkaido (SA, KT), one in Chubu (GC), and two in Kyushu (QB, QD).

Antimicrobial susceptibility was determined using the agar dilution method in accordance with the Clinical Laboratory Standard Institute (CLSI) guidelines (11); ampicillin (ABPC; 32 μg mL^−1^), cefazolin (CEZ; 32 μg mL^−1^), ceftiofur (CTF; 8 μg mL^−1^), dihydrostreptomycin (DSM; 32 μg mL^−1^), kanamycin (KM; 64 μg mL^−1^), gentamycin (GM; 16 μg mL^−1^), bicozamycin (BCM; 128 μg mL^−1^), oxytetracycline (OTC; 16 μg mL^−1^), colistin (CL; 16 μg mL^−1^), chloramphenicol (CP; 32 μg mL^−1^), nalidixic acid (NA; 32 μg mL^−1^), enrofloxacin (ERFX; 2 μg mL^−1^), and trimethoprim (TMP; 16 μg mL^−1^) (11, 43). These agents were selected from the Japanese Veterinary Antimicrobial Resistance Monitoring System (http://www.maff.go.jp/nval/tyosa_kenkyu/taiseiki/index.html). The antimicrobial resistance breakpoint as the minimum inhibitory concentration for antimicrobial agents was used according to the CLSI criteria. *E. coli* ATCC 25922, *Staphylococcus aureus* ATCC 29213, *Enterococcus faecalis* ATCC 29212, and *Pseudomonas aeruginosa* ATCC 27853 were used as quality control strains.

### AMR genotype analysis

We assessed the isolates for the following AMR genes (16, 21, 22, 26, 30, 31, 34, 43): (i) resistance to beta-lactam agents (ABPC, CEZ, and CFT): *bla*_TEM_, *bla*_SHV_, *bla*_OXA_, *bla*_CTX-M_, and *bla*_CMY_; (ii) resistance to aminoglycosides (DSM, GM, and KM): *strA*, *strB*, *aadB*, *aacC2*, *aac(3)-IV*, *aphA1*, *aphA2*, *aadD*, *aphA3*, *aphA/aph(3′)-Id*, *aphA7*, *aphAI-IAB*, *Kn*, and *Kan*; (iii) resistance to tetracycline (OTC): *tetA*, *tetB*, *tetC*, *tetD*, *tetE*, *tetG*, *tetY*, and *tetS*; (iv) resistance to CP: *catI*, *catII*, *catIII*, *floR*, and *cmlA*; and (v) resistance to TMP: *dhfrI*, *dhfrV*, *dhfrVII*, *dhfrIX*, *dhfrXIII*, *dfrA12*, *dhfrXVII*, and *dhfrXII*.

The presence of AMR genes were determined by PCR with the total DNA of each of the 45 strains as the template using primers reported in previous studies (primers and original studies are listed in Table S1). GoTaq^®^ Flexi DNA Polymerase (Promega, Madison, WI, USA) was used for the PCR detection, and amplification was performed under the following conditions: 30 s at 95°C, followed by 30 cycles of 95°C for 15 s, an appropriate annealing temperature (described in Table S1) for 30 s, 72°C for 1 min, and a final extension at 72°C for 5 min. A 100-bp DNA ladder (Sigma-Aldrich, St. Louis, MO, USA) was used as the molecular marker. When an amplicon was obtained, the nucleotide sequence was determined and used to prepare AMR gene-specific hybridization probes. These nucleotide sequences showed 99–100% identity with known antibiotic resistance genes (reference genes). The presence of the PCR-detected AMR genes was then further confirmed by colony hybridization using specific probes. Probe labeling and hybridization were performed by the Alkphos Direct Labelling and Detection System (GE Healthcare, Buckinghamshire, UK) following the manufacturer’s instructions with a hybridization temperature of 55°C and washing twice at 55°C. The hybridization signal was detected by the CDP-Star^TM^ Detection Reagent (GE Healthcare).

### Plasmid detection and size estimation

Detection of the plasmid and determining its size were achieved by two methods (14, 37). Regarding small plasmids (2–16 kb), *E. coli* cells were lysed by the conventional alkaline method and the extracted plasmids were then separated by 0.7% agarose gel electrophoresis with 0.5× Tris-acetate-EDTA buffer (37) and the super-coiled DNA Ladder (Invitrogen/Life Technologies, Carlsbad, CA, USA) as a molecular marker. Regarding large plasmids (>16 kb), pulsed-field gel electrophoresis (PFGE) was performed with S1-nuclease (Invitrogen/Life Technologies)-digested plasmid DNA (see below for PFGE conditions) (14). The size of the plasmid was calculated using a standard curve of the logarithmic approximation obtained from the distance between electrophoretic image bands and the DNA size marker.

### Plasmid type analysis

At least 18 types of plasmid incompatibility have been reported in *E. coli*. These incompatibility types have been distinguished by specific nucleotide sequences (6) and identified as IncHI1, HI2, I1, X, L/M, N, FIA, FIB, W, Y, P, FIC, A/C, T, FIIAs, F, K, and B/O. In the present study, the incompatibility type was firstly detected by PCR with the total DNA of each of the 45 strains as the template using specific primers (Table S1) (6). GoTaq^®^ Flexi DNA Polymerase (Promega) was used for the PCR detection, and amplification was performed under the following conditions: 30 s at 95°C, followed by 30 cycles of 95°C for 15 s, an appropriate annealing temperature (described in Table S1) for 30 s, 72°C for 1 min, and a final extension at 72°C for 5 min. A 100-bp DNA ladder (Sigma-Aldrich) was used as the molecular marker. When an amplicon was obtained, the nucleotide sequence was determined and used to pre pare incompatibility-specific hybridization probes. These nucleotide sequences showed 98–100% identity with known incompatibility plasmid sequences (reference plasmid). The incompatibility type of each plasmid was determined by Southern hybridization with incompatibility-specific probes against the PFGE-separated plasmids of the 45 strains. Probe preparation and hybridization were performed as described in the AMR genotype analysis section.

### Pulse-field gel electrophoresis (PFGE)

PFGE was performed with a CHEF-DRII system (Bio-Rad Laboratories, Hercules, CA) as follows. (i) PFGE for large plasmids (>16 kb): Agarose plugs were prepared with a cell suspension at A_610_ 1.6–1.7 according to the Pulse Net Protocol (http://www.pulsenetinternational.org/Pages/default.aspx). Plugs were digested with S1-nuclease (Invitrogen, 5 units) for 10 min at 37°C. Electrophoresis was performed with pulse times from 1 to 25 s, voltage 6 V cm^−1^, angle orientation 120°, and a run time of 14.5 h in 1% SeaKem Gold agarose (Cambrex, Rockland, ME) with 0.5× Tris-borate-EDTA buffer at 14°C. Low-range PFG markers (New England Biolabs, Schwalbach, Germany) were used as size markers. (ii) PFGE for RFLP: Agarose plugs were prepared with a cell suspension at A_610_ 1.00. Plugs were digested with 50 U *Xba*I for 2 h at 37°C. Electrophoresis was performed with pulse times of 2.2 to 63.8 s, voltage 6 V cm^−1^, angle orientation 120°, and a run time of 18.0 h in 1% SeaKem Gold agarose with 0.5× Tris-borate-EDTA buffer at 14°C. Lambda Ladder PFG markers (New England Biolabs) were used as size markers.

## Results

### Chromosome phylotypes of 45 *E. coli* isolates

**Fig. 1:**
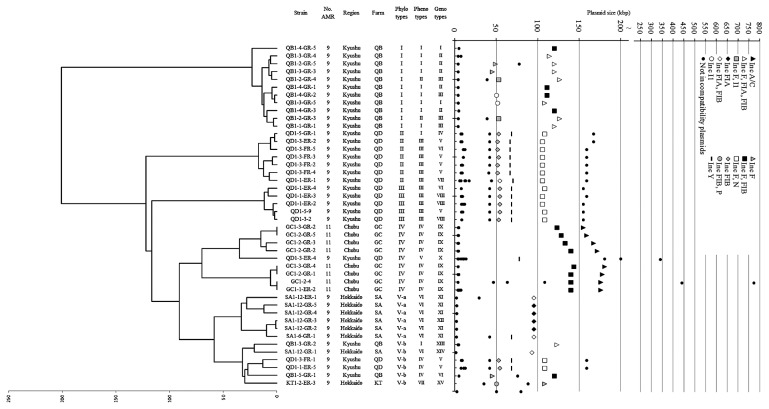
Dendrogram of the relationship between phylotypes obtained from PFGE patterns and plasmid types in 44 *E. coli* isolates. The dendrogram and plasmid profiles are displayed in parallel to show the relationship between the phylotype (left) and plasmid type (right) in each isolate. The phylotypes were obtained from the RFLP patterns of *Xba*I-digested whole genomes and were classified into five groups at a divergence value of 70; these were named phylotypes I to V, and phylotype V was further divided into V-a or V-b at a divergence value of 58. Plasmids were separated using alkali lysis and PFGE followed by S1-nuclease digestion. Plasmid symbols indicate the incompatibility type. Symbols with multiple incompatibility designations indicated that multiple probes of several incompatibility groups hybridized on the same or same-sized plasmid(s). Strains and AMR resistances are listed in Table 1.

Forty-five isolates resistant to at least nine agents were selected from 3,147 *E. coli* strains isolated from the feces of beef cattle in Hokkaido, Chubu, and Kyushu, Japan (43). Phylotype analyses were performed with *Xba*I-RFLP followed by cluster analysis. The genomic DNA of each isolate yielded 13–27 *Xba*I fragments on PFGE, and 83 different-sized fragments were obtained. The RFLP profiles were subjected to cluster analysis to construct a phylogenetic dendrogram (see Materials and Methods). An RFLP profile could not be obtained for isolate SA1-4-1 (resistant to nine agents) despite repeated trials. This may have been because of the ease of lysis and instant DNA degradation of the isolate. Therefore, the dendrogram was built with the profiles of 44 isolates (Fig. 1). Cluster analysis indicated that the overall average divergence value was 25. The values of the closest and most distant were zero and 201, respectively. The isolates were clearly classified into five groups at a divergence value of 70, and were designated as phylotypes I to V (Fig. 1). Members in phylotype I were isolated from the same farm (QB) in Kyushu. Members in phylotypes II and III were isolated from the same farm (QD) in Kyushu and were distant at a divergence value of 122. This indicated that farm QD was occupied by phylotype groups II and III. Phylotype IV was composed of nine isolates, eight from farm GC in Chubu and one from farm QD in Kyushu. The unique isolate from farm QD (QD1-3-ER-4) was resistant to nine agents, while the others were resistant to 11 agents. Phylotype V consisted of 12 isolates, could be subclassified into two groups at a divergence value of 58, and the members in each subgroup were within a divergence value of 32. Members in the first subgroup (V-a) consisted of six isolates from farm SA in Hokkaido. On the other hand, the origin of the members in the second subgroup (V-b) varied; one each from farms SA and KT in Hokkaido, and two each from farms QB and QD in Kyushu.

### AMR phenotype analysis of 45 *E. coli* isolates

Thirty-seven of the 45 isolates showed antibiotic resistance to nine agents (9-AMR isolates); the remainder were resistant to 11 agents (11-AMR isolates). The AMR phenotypes (AMR susceptibility) for 13 antibiotics were determined by the agar dilution method. All 45 isolates were resistant to ABPC, DSM, OTC, NA, and TMP (Table 1). Resistance was detected for CEZ (number of isolates; n = 44), GM (n = 43), CTF (n = 34), ERFX (n = 32), KM (n = 22), BCM (n = 10), and CP (n = 10). Only one isolate was resistant to CL (SA1-4-1). The 11-AMR isolates shared an identical profile of resistance to ABPC, CEZ, CTF, DSM, GM, BCM, OTC, CP, NA, ERFX, and TMP. They were grouped together into phenotype group IV and were isolated from the same farm in Chubu (GC). In contrast, the 9-AMR isolates were divided into seven phenotype groups (I–III, V–VIII). These were isolated from Hokkaido and Kyushu. The AMR profile of each isolate and phenotype grouping are listed in Table 1. The AMR phenotype generally correlated with the phylotype. Members in phenotype II fell into phylotype I, phenotype III fell into phylotype II or III, and phenotype VI fell into phylotype V-a.

### AMR genotype analysis of 45 *E. coli* isolates

To determine the AMR genotype, we assessed each isolate for the presence of 40 AMR genes by PCR amplification and confirmed our results using colony and Southern hybridization. Since the nucleotide sequences of resistance genes for BCM and CL have not yet been identified, these genes were not assessed. Resistance genes for quinolone antibiotics (NA and ERFX) were not assessed because resistance to these drugs is derived from DNA gyrase mutations rather than by specific resistance genes and enzymes. Of the 40 AMR genes tested, 17 were detected; each isolate contained 7–10 genes. The other 23 AMR genes were not detected. The AMR genes and genotype grouping are listed in Table 1; the number of isolates harboring the given AMR genes is listed in Table 2. The most common AMR gene in this study was *strA (*an aminoglycoside resistance gene), which was detected in all 45 isolates. The other major AMR genes (detected in over 30 isolates) were *strB* (44 isolates), *bla*_CMY_ (39 isolates), *aacC2* and *tetB* (35 isolates), and *bla*_TEM_ (31 isolates). The least common AMR gene was *dhfrI* (8 isolates). Isolates were divided into 16 genotype groups based on the combination of resistance genes (Table 1). The 11-AMR isolates were classified into the same genotype group (Genotype group IX), whereas the 9-AMR isolates were divided into 15 genotype groups (Genotype groups I–VIII and X–XVI). We expected the AMR phenotype to correspond to the AMR genotype in each isolate. However, exceptions were found in 16 isolates. For example, seven isolates were susceptible to CP even though they harbored the *catI* gene. Although nine isolates were resistant to TMP, none of the corresponding resistance genes were detected.

AMR genotype groups IX and XI correlated with phylotypes I and V-a, respectively. However, the other AMR genotype groups did not match the phylotype groups. Each phylotype group included 3–5 AMR genotype groups.

### Plasmid type analysis of 45 *E. coli* isolates

Plasmid sizes were determined by conventional gel electrophoresis and PFGE with appropriate DNA size markers. All isolates carried plasmids of various sizes, ranging between 2 and 774 kb. A total of 210 individual plasmids were identified and divided into 58 classes by size (Fig. 1). All isolates carried at least two plasmids including large ones (>16 kb); two isolates carried ten plasmids each. To date, at least 18 incompatibility type plasmids have been sequenced and reported (6). In this study, these incompatibility types were defined as “typical incompatibility group” plasmids. Eight of the 18 incompatibility types were identified in the 45 isolates, including IncF, FIA, FIB, I1, N, P, Y, and A/C (Table 1, 3). In addition to these typical incompatibility group plasmids, we also detected plasmids for which a typical incompatibility group could not be determined. These unidentified plasmids were defined as the “unidentified group”. The 210 individual plasmids comprised 89 typical incompatibility group plasmids (46 to 181 kb in sizes) and 121 unidentified group plasmids (2 to 774 kb). Plasmids were classified into 74 varieties according to size and incompatibility type (Fig. 1). Each isolate carried one to 5 different incompatibility type plasmids. The most common typical incompatibility group was IncFIB, found in 39 isolates, followed by IncF, in 37 isolates, and IncFIA, in 16 isolates. One exceptional isolate (QD1-3-ER-4) had none of these, but carried an IncY plasmid. The 11-AMR isolates carried typical incompatibility groups IncF, FIB, and A/C, and were grouped into Inc type group VIII (Table 1). The IncA/C type, known for multiple-antibiotic resistance, was detected in 11-AMR isolates, but was not in 9-AMR isolates. The 9-AMR isolates were divided into 13 groups (Inc type groups I–VII, IX–XIV) according to their combination of incompatibility types (Table 1).

### AMR genes on each type plasmid

Southern hybridization was performed with 17 AMR gene-specific probes against PFGE-separated plasmid DNA in order to identify the AMR genes on each type plasmid. The number of AMR genes on individual plasmids ranged from 1–9. Various combinations of AMR genes were observed, as listed in Table 3. The IncFIB, F, and FIA plasmids covered various AMR genes, carrying 15, 14, and 10 genes, respectively. IncY, N, A/C, and I1 covered 1–8 AMR genes. The IncP plasmid identified in one isolate (KT1-2-ER-3) harbored no AMR genes. In addition to these typical incompatibility group plasmids, the unidentified group also contained AMR genes: nine varieties were detected in the present study. Some AMR genes were also found to be widely distributed. For example, *bla*_TEM_ was distributed in IncFIB, F, FIA, A/C, and I1. The other example, *tetA*, was distributed in IncFIB, F, N, A/C, and the unidentified group.

The 210 detected plasmids were classified into 92 groups by size, incompatibility type, and AMR genes (Table S2). Twenty-eight groups contained one AMR gene and 41 groups contained more than two. The 11-AMR isolates contained IncA/C plasmids carrying a maximum of seven genes, whereas the 9-AMR isolates contained IncFIA and FIB plasmids carrying a maximum of nine genes.

### Relationship between chromosome phylotypes and plasmid types

To characterize the relationship between host *E. coli* and the plasmid type, the phylotype dendrogram and plasmid profiles were displayed in parallel (Fig. 1). Each isolate of phylotype I contained a similar plasmid set composed of six plasmid types. Although members in phylotypes II and III were distant at a divergence value of 122, their plasmid types were highly similar. This result indicated that plasmids or parts of plasmids may be transferred among phylotypes II and III. The plasmid types detected within members in phylotype IV were similar, except for QD1-3-ER-4. This was a 9-AMR isolate with a unique AMR phenotype, AMR genes, and plasmid type. Members in phylotype V-a shared a similar plasmid type, except for IncY in isolate SA1-6-GR-1. The isolates in phylotype V-b obtained from four different farms in Hokkaido and Kyushu were divided into five plasmid types. QD1-3-FR-1 and QD1-1-ER-5 in phylotype V-b were distant from phylotype II and III from QD, although their plasmid types were similar. QB1-5-GR-1 in phylotype V-b was distant from phylotype I from QB, although their plasmid types were similar.

## Discussion

In the present study, we analyzed the phenotypic and genotypic traits of 45 multiple-antibiotic resistant *E. coli* isolates based on the chromosome phylotype (PFGE pattern of the digested genome), AMR phenotype (pattern of multiple-antibiotic resistance), AMR genotype (the presence or absence of 40 AMR genes), and plasmid type (incompatibility type), and attempted to link these traits in order to characterize the mechanism by which multiple-antibiotic resistant *E. coli* are distributed in livestock farms.

### Chromosome phylotypes

Multiple-antibiotic resistant *E. coli* were classified into five chromosome phylotypes (type V was further subgrouped into V-a and V-b; Fig. 1). These chromosome phylotypes were closely linked to the farms from which they were isolated, *i.e.* the members in phylotypes I, II, III, IV, and V-a were mainly isolated from farms QB, QD, QD, GC, and SA, respectively, which suggested that each phylotype may be endemic to a specific area (*i.e.* livestock farms). Many previous studies on *E. coli* phylotype analyses have so far shown that the cross-contamination of phylotypes over a wide area frequently occurs as a result of animal transport (29, 42). The results of our study indicated that each phylotype was generally well conserved in each district. Nonetheless, notable exceptions were found: 1) isolates within phylotype V-b were widespread in QB (Kyushu), QD (Kyushu), SA (Hokkaido), and KT (Hokkaido); 2) farm QB harbored not only phylotype I (as the dominant phylotype), but also V-b; 3) farm QD harbored not only phylotypes II and III (as the dominant phylotypes), but also IV and V-b. The cross-over of such phylotypes may have been because of livestock import and export for feeding and breeding.

### Antimicrobial resistance phenotype and genotype analyses

We investigated the AMR phenotypes and genotypes of 45 *E. coli* isolates. These isolates were classified into eight AMR phenotypes (phenotype group I–VIII, Table 1); however, genotype analysis revealed 16 AMR patterns (genotype group I–XVI, Table 1). AMR phenotyping was consistent with chromosomal phylotyping and, thus, correlated to the farms from which they were isolated (Fig. 1 and Table 1). However, AMR genotyping revealed a different narrative; AMR genotypes were widespread across various AMR phylotypes, with no correlation being observed between the two. AMR phenotyping is compelling because it is based on resistance to a given antibiotic at specified concentrations. In contrast, AMR genotyping relies on the presence or absence of antibiotic resistance genes that have been reported previously. Therefore, it is likely that this method of genotyping will overlook genes that have significant mismatches for PCR amplification or are unknown, even though their presence is evident. We were unable to detect several resistance genes that should have been present. For example, although strain QB1-3-GR-4 is resistant to TMP, no TMP gene was detected.

### Redundancy of resistance genes

More than two AMR genes conferring resistance to the same antibiotics were found in a single isolate—*bla*_TEM_ and *bla*_CMY_, *bla*_CTX-M_ and *bla*_CMY_, *strA* and *strB*, and *dhfrXIII* and *dfrA12* (Table 2). Similar genetic redundancies have been reported elsewhere (3, 34). The presence of *strA* and *strB* provides additive streptomycin resistance. Chuou and Jones demonstrated high-level streptomycin resistance in the presence of *strA* and *strB* (10). We identified tetracycline-resistant isolates carrying *tetA* and *tetB* even though a previous study suggested that these genes could not co-exist on a plasmid, and a negative correlation was found in *E. coli (*4). This negative relationship may be related to the correlation between the replication genes of the incompatibility plasmid and the tetracycline resistance determinant (27). Our results appeared to be an exceptional case; thus, further studies to understand the negative relationship between these two parameters are warranted.

### Plasmid types and AMR genes

We tested for the presence of 18 known incompatibility types and identified eight typical incompatibility group plasmids. In addition, 40 known AMR genes were tested and 17 were confirmed. Moreover, the localization of the 17 AMR genes on the plasmid was determined in the present study. Some known incompatibility type plasmids have been found to frequently carry AMR genes; however, these studies focused on a limited number of typical incompatibility group plasmids and AMR genes (7, 9, 12, 19, 32, 39). In our study, 95.5% of the typical incompatibility group plasmids contained 1–9 AMR genes. These results confirmed the link between the plasmid type and AMR genes. IncA/C plasmids are known to be multiple-antibiotic-resistant plasmids with a conserved backbone and variable accessory components such as AMR-encoding elements (5, 15). The whole nucleotide sequences of five IncA/C plasmids have been determined and shown to carry eight or twelve AMR genes (5, 15). In our study, five AMR genes were confirmed on an IncA/C plasmid. Moreover, IncFIA and FIB plasmids carrying nine AMR genes were detected. To the best of our knowledge, IncFIA or FIB plasmids harboring nine or more AMR genes have not been reported elsewhere (7, 33, 39). In contrast, other plasmids contained 1–6 AMR genes. AMR genes on plasmids have largely been investigated on typical incompatibility group plasmids and few have been reported on unidentified group plasmids (7, 9, 12, 19, 32, 39). However, unidentified group plasmids containing up to six AMR genes were detected in the present study. Comprehensive plasmid-AMR gene relationship analysis of typical incompatibility groups and the unidentified group is needed to understand the distributions and spreading mechanisms of AMR genes.

### Comparison of plasmid type and chromosome phylotype

Plasmid incompatibility, in terms of the relationship between host phylotype and plasmids that confer multidrug resistance, has not yet been examined. The plasmid type of each isolate was associated with the phylotype, which indicated that the plasmid type also reflects the farms from which they were isolated; in other words, each local farm had its own plasmid type. Some exceptions were found. For example, the plasmid type of two isolates in phylotype V-b (QD1-3-FR-1 and QD1-1-ER-5) was similar to that in phylotypes II and III (Fig. 1). The plasmid type of QB1-5-GR-1 (phylotype V-b) was also similar to that of phylotype I. In contrast, V-b contained different varieties of phenotypes, plasmid types, and farm locations. Therefore, members in phylotype V-b were distributed in several farms and contained the farm’s respective plasmids, which were shared in multiple phylotype groups. These results demonstrated that plasmid sets could be transferred between phylotypes, perhaps as a result of the commercial custom of transferring young cattle to different farms via livestock markets.

## Conclusion

In the present study, we investigated 45 multiple-antibiotic-resistant *E. coli* strains from five beef cattle farms and demonstrated their chromosome phylotype diversity at the inter-farm level. We also showed inter- and intra-farm level diversity in the AMR genotype. This may have been due to the cross-contamination of AMR genes mediated by animal transportation. The phylotype diversity of AMR strains between livestock farms has been reported previously (29, 42). The coexistence of AMR strains with different AMR genes may promote the emergence of more complex multi-antibiotic resistance strains via plasmid transfer and exchange. The types and combinations of AMR genes were demonstrated using 9- and 11-AMR strains in the present study. An investigation of other AMR strains (*i.e.* 8-AMR or less) will help define the distribution of AMR genes, their mechanisms, and route of dispersal. The results of our study provide fundamental information of the prevalence and diversity of AMR strains for epidemiological studies and important information for identifying or predicting the emergence of multi-antibiotic resistance microorganisms.

## Supplementary Information



## Figures and Tables

**Table 1:** Phylotypes, AMR phenotypes, AMR genotypes, and incompatibility plasmid types

Strains	Region	Farm	Phylotypes	AMR phenotypes		AMR genotypes		Incompatibility plasmid (Inc) types	
		
				Phenotypes showed^a^^b^	Phenotype group	AMR genes detected^c^	Genotype group	Inc plasmid detected	Inc type group
QB1-4-GR-5	Kyushu	QB	I	CEZ, CTF, KM, GM	I	*bla*_TEM_*, bla*_CMY_*, strA, strB, aacC2, aphA1, aphAI-IAB, tetB, tetC, dhfrXIII*	I	F, FIB	I
QB1-3-GR-4	Kyushu	QB	I	CEZ, CTF, KM, GM	I	*bla*_TEM_*, bla*_CMY_*, strA, strB, aacC2, aphA1, aphAI-IAB, tetB, tetC*	II	F, FIA, FIB	II
QB1-2-GR-5	Kyushu	QB	I	CEZ, CTF, KM, GM	I	*bla*_TEM_*, bla*_CMY_*, strA, strB, aacC2, aphA1, aphAI-IAB, tetB, tetC*	II	F, FIA, FIB	II
QB1-3-GR-3	Kyushu	QB	I	CEZ, CTF, KM, GM	I	*bla*_TEM_*, bla*_CMY_*, strA, strB, aacC2, aphA1, aphAI-IAB, tetB, tetC*	II	F, FIA, FIB	II
QB1-2-GR-4	Kyushu	QB	I	CEZ, KM, GM, BCM	II	*bla*_TEM_*, bla*_CMY_*, strA, strB, aacC2, aphA1, aphAI-IAB, tetB*	III	F, FIA, FIB, I1	III
QB1-4-GR-1	Kyushu	QB	I	CEZ, CTF, KM, GM	I	*bla*_TEM_*, bla*_CMY_*, strA, strB, aacC2, aphA1, aphAI-IAB, tetB, tetC*	II	F, FIB	IV
QB1-4-GR-2	Kyushu	QB	I	CEZ, CTF, KM, GM	I	*bla*_TEM_*, bla*_CMY_*, strA, strB, aacC2, aphA1, aphAI-IAB, tetB*	III	F, FIB, I1	V
QB1-3-GR-5	Kyushu	QB	I	CEZ, CTF, KM, GM	I	*bla*_TEM_*, bla*_CMY_*, strA, strB, aacC2, aphA1, aphAI-IAB, tetB, tetC, dhfrXIII*	I	F, FIA, FIB, I1	III
QB1-4-GR-3	Kyushu	QB	I	CEZ, CTF, KM, GM	I	*bla*_TEM_*, bla*_CMY_*, strA, strB, aacC2, aphA1, aphAI-IAB, tetB, tetC*	II	F, FIB	IV
QB1-2-GR-3	Kyushu	QB	I	CEZ, KM, GM, BCM	II	*bla*_TEM_*, bla*_CMY_*, strA, strB, aacC2, aphA1, aphAI-IAB, tetB*	III	F, FIA, FIB, I1	III
QB1-1-GR-1	Kyushu	QB	I	CEZ, CTF, KM, GM	I	*bla*_TEM_*, bla*_CMY_*, strA, strB, aacC2, aphA1, aphAI-IAB, tetB*	III	F, FIA, FIB	II
QD1-5-GR-1	Kyushu	QD	II	CEZ, CTF, KM, GM	I	*bla*_TEM_*, bla*_CMY_*, strA, strB, aacC2, aphA1, aphAI-IAB, tetB, tetC, dfrA12*	IV	F, FIB, N, Y	VI
QD1-3-ER-2	Kyushu	QD	II	CEZ, CTF, GM, ERFX	III	*bla*_CTX-M_*, bla*_CMY_*, strA, strB, aacC2, tetA, tetB, tetC, dhfrXIII, dfrA12*	V	F, FIB, N, Y	VI
QD1-3-FR-5	Kyushu	QD	II	CEZ, CTF, GM, ERFX	III	*bla*_CTX-M_*, bla*_CMY_*, strA, strB, aacC2, tetA, tetB, dhfrXIII, dfrA12*	VI	F, FIB, N, Y	VI
QD1-3-FR-3	Kyushu	QD	II	CEZ, CTF, GM, ERFX	III	*bla*_CTX-M_*, bla*_CMY_*, strA, strB, aacC2, tetA, tetB, tetC, dhfrXIII, dfrA12*	V	F, FIB, N, Y	VI
QD1-3-FR-2	Kyushu	QD	II	CEZ, CTF, GM, ERFX	III	*bla*_CTX-M_*, bla*_CMY_*, strA, strB, aacC2, tetA, tetB, tetC, dhfrXIII, dfrA12*	V	F, FIB, N, Y	VI
QD1-3-FR-4	Kyushu	QD	II	CEZ, CTF, GM, ERFX	III	*bla*_CTX-M_*, bla*_CMY_*, strA, strB, aacC2, tetA, tetB, tetC, dhfrXIII, dfrA12*	V	F, FIB, N, Y	VI
QD1-1-ER-1	Kyushu	QD	II	CEZ, CTF, GM, ERFX	III	*bla*_CTX-M_*, strA, strB, aacC2, tetA, tetB, dfrA12*	VII	F, FIA, FIB, N, Y	VII
QD1-1-ER-4	Kyushu	QD	III	CEZ, CTF, GM, ERFX	III	*bla*_CTX-M_*, bla*_CMY_*, strA, strB, aacC2, tetA, tetB, dhfrXIII, dfrA12*	VI	F, FIB, N, Y	VI
QD1-1-ER-3	Kyushu	QD	III	CEZ, CTF, GM, ERFX	III	*bla*_CTX-M_*, bla*_CMY_*, strA, strB, aacC2, tetA, tetB, tetC, dfrA12*	VIII	F, FIB, N, Y	VI
QD1-1-ER-2	Kyushu	QD	III	CEZ, CTF, GM, ERFX	III	*bla*_CTX-M_*, bla*_CMY_*, strA, strB, aacC2, tetA, tetB, tetC, dfrA12*	VIII	F, FIB, N, Y	VI
QD1-5-9	Kyushu	QD	III	CEZ, CTF, GM, ERFX	III	*bla*_CTX-M_*, bla*_CMY_*, strA, strB, aacC2, tetA, tetB, tetC, dhfrXIII, dfrA12*	V	F, FIB, N, Y	VI
QD1-3-2	Kyushu	QD	III	CEZ, CTF, GM, ERFX	III	*bla*_CTX-M_*, bla*_CMY_*, strA, strB, aacC2, tetA, tetB, tetC, dfrA12*	VIII	F, FIB, N, Y	VI
GC1-3-GR-2	Chubu	GC	IV	CEZ, CTF, GM, BCM, CP, ERFX	IV	*bla*_TEM_*, bla*_CMY_*, strA, strB, tetA, tetC, floR, dhfrI*	IX	F, FIB, A/C	VIII
GC1-2-GR-5	Chubu	GC	IV	CEZ, CTF, GM, BCM, CP, ERFX	IV	*bla*_TEM_*, bla*_CMY_*, strA, strB, tetA, tetC, floR, dhfrI*	IX	F, FIB, A/C	VIII
GC1-2-GR-3	Chubu	GC	IV	CEZ, CTF, GM, BCM, CP, ERFX	IV	*bla*_TEM_*, bla*_CMY_*, strA, strB, tetA, tetC, floR, dhfrI*	IX	F, FIB, A/C	VIII
GC1-2-GR-2	Chubu	GC	IV	CEZ, CTF, GM, BCM, CP, ERFX	IV	*bla*_TEM_*, bla*_CMY_*, strA, strB, tetA, tetC, floR, dhfrI*	IX	F, FIB, A/C	VIII
QD1-3-ER-4	Kyushu	QD	IV	CEZ, GM, CP, ERFX	V	*bla*_TEM_*, strA, strB, aacC2, tetA, tetC, catI, dfrA12*	X	Y	IX
GC1-3-GR-4	Chubu	GC	IV	CEZ, CTF, GM, BCM, CP, ERFX	IV	*bla*_TEM_*, bla*_CMY_*, strA, strB, tetA, tetC, floR, dhfrI*	IX	F, FIB, A/C	VIII
GC1-2-GR-1	Chubu	GC	IV	CEZ, CTF, GM, BCM, CP, ERFX	IV	*bla*_TEM_*, bla*_CMY_*, strA, strB, tetA, tetC, floR, dhfrI*	IX	F, FIB, A/C	VIII
GC1-2-4	Chubu	GC	IV	CEZ, CTF, GM, BCM, CP, ERFX	IV	*bla*_TEM_*, bla*_CMY_*, strA, strB, tetA, tetC, floR, dhfrI*	IX	F, FIB, A/C	VIII
GC1-1-ER-2	Chubu	GC	IV	CEZ, CTF, GM, BCM, CP, ERFX	IV	*bla*_TEM_*, bla*_CMY_*, strA, strB, tetA, tetC, floR, dhfrI*	IX	F, FIB, A/C	VIII
SA1-12-ER-1	Hokkaido	SA	V-a	CEZ, KM, GM, ERFX	VI	*bla*_TEM_*, bla*_CMY_*, strA, strB, aacC2, aphA1, aphAI-IAB, tetB, catI, dhfrVII*	XI	FIA, FIB	X
SA1-12-GR-5	Hokkaido	SA	V-a	CEZ, KM, GM, ERFX	VI	*bla*_TEM_*, bla*_CMY_*, strA, strB, aacC2, aphA1, aphAI-IAB, tetB, catI, dhfrVII*	XI	FIA	XI
SA1-12-GR-4	Hokkaido	SA	V-a	CEZ, KM, GM, ERFX	VI	*bla*_TEM_*, bla*_CMY_*, strA, strB, aacC2, aphA1, aphAI-IAB, tetB, catI, dhfrVII*	XI	FIA	XI
SA1-12-GR-3	Hokkaido	SA	V-a	CEZ, KM, GM, ERFX	VI	*bla*_TEM_*, strA, aacC2, aphA1, aphAI-IAB, tetB, catI, dhfrVII*	XII	FIA	XI
SA1-12-GR-2	Hokkaido	SA	V-a	CEZ, KM, GM, ERFX	VI	*bla*_TEM_*, bla*_CMY_*, strA, strB, aacC2, aphA1, aphAI-IAB, tetB, catI, dhfrVII*	XI	FIA	XI
SA1-6-GR-1	Hokkaido	SA	V-a	CEZ, KM, GM, ERFX	VI	*bla*_TEM_*, bla*_CMY_*, strA, strB, aacC2, aphA1, aphAI-IAB, tetB, catI, dhfrVII*	XI	FIA, FIB, Y	XII
QB1-3-GR-2	Kyushu	QB	V-b	CEZ, CTF, KM, GM	I	*bla*_TEM_*, strA, strB, aacC2, aphA1, aphAI-IAB, tetB, dfrA12*	XIII	F, FIA, FIB	II
SA1-12-GR-1	Hokkaido	SA	V-b	CEZ, KM, GM, ERFX	VI	*bla*_TEM_*, strA, strB, aacC2, aphA1, aphAI-IAB, tetB, catI, dhfrVII*	XIV	FIA, FIB	X
QD1-3-FR-1	Kyushu	QD	V-b	CEZ, CTF, GM, ERFX	IV	*bla*_CTX-M_*, bla*_CMY_*, strA, strB, aacC2, tetA, tetB, tetC, dhfrXIII, dfrA12*	V	F, FIB, N, Y	VI
QD1-1-ER-5	Kyushu	QD	V-b	CEZ, CTF, GM, ERFX	IV	*bla*_CTX-M_*, bla*_CMY_*, strA, strB, aacC2, tetA, tetB, tetC, dhfrXIII, dfrA12*	V	F, FIB, N, Y	VI
QB1-5-GR-1	Kyushu	QB	V-b	CEZ, CTF, GM, ERFX	IV	*bla*_CTX-M_*, bla*_CMY_*, strA, strB, aacC2, tetA, tetB, dhfrXIII, dfrA12*	VI	F, FIB	I
KT1-2-ER-3	Hokkaido	KT	V-b	CEZ, CTF, KM, ERFX	VII	*bla*_TEM_*, strA, strB, aphA1, aphAI-IAB, tetA, tetC, dhfrVII*	XV	F, FIB, P	XIII
SA1-4-1	Hokkaido	SA	Not shown	KM, CL, CP, ERFX	VIII	*bla*_TEM_*, bla*_CMY_*, strA, strB, aphA1, aphAI-IAB, tetB, catI, floR, dhfrVII*	XVI	F, I1	XIV
Total numbers			6 types		8 types		16 types		14 types

a ABPC, ampicillin; CEZ, cefazolin; CTF, ceftiofur; DSM, dihydrostreptomycin; KM, kanamycin; GM, gentamicin; BCM, bicozamycin; OTC, oxytetracycline; CL, colistin; CP, chloramphenicol; NA, nalidixic acid; ERFX, enrofloxacin; TMP, trimethoprim.

b All isolates had resistance to ABPC, DSM, OTC, NA, and TMP. These antimicrobial agents weren’t described above.

c *bla*_TEM_, *bla*_CTX-M_ and *bla*_CMY_, beta-lactams resistance genes; *strA* and *strB*, DSM resistance gene; *aacC2*, GM resistance gene; *aphA1* and *aphAI-IAB*, KM resistance gene; *tetA*, *tetB* and *tetC*, OTC resistance gene; *catI* and *floR*, CP resistance gene; *dhfrI*, *dhfrVII*, *dhfrXIII* and *dfrA12*, TMP resistance gene.

**Table 2:** AMR genes in 45 multi-drug-resistant *E. coli* isolates

Resistance genes^a^		n^b^	Number of isolates possessed the gene
Beta-lactams (ABPC, CEZ, CTF)		45	
	*bla*_TEM_		31
	*bla*_SHV_		0
	*bla*_OXA_		0
	*bla*_CTX-M_		14
	*bla*_CMY_		39
	*bla*_TEM_ + *bla*_CMY_		26
	*bla*_CTX-M_ + *bla*_CMY_		13
Aminoglycosides			
DSM		45	
	*strA*^b^		45
	*strB*^b^		44
	*strA + strB*		44
GM		43	
	*aadB*^c^		0
	*aacC2*^c^		35
	*aac(3)-IV*^c^		0
KM		22	
	*aphA1*^d^		22
	*aphA2*^d^		0
	*aadD*^d^		0
	*aphA3*^d^		0
	*aphA/aph(3′)-Id*^d^		0
	*aphA7*^d^		0
	*aphAI-IAB*^d^		22
	*Kn*^d^		0
	*Kan*^d^		0
	*aphA1 + aphAI-IAB*		22
OTC		45	
	*tetA*		24
	*tetB*		35
	*tetC*		28
	*tetD*		0
	*tetE*		0
	*tetG*		0
	*tetY*		0
	*tetS*		0
	*tetA + tetB*		14
	*tetB + tetC*		18
	*tetA + tetC*		20
	*tetA + tetB + tetC*		10
CP		10	
	*catI*		9
	*catII*		0
	*catIII*		0
	*floR*		9
	*cmlA*		0
	*catI + floR*		1
TMP		45	
	*dhfrI*		8
	*dhfrV*		0
	*dhfrVII*		9
	*dhfrIX*		0
	*dhfrXIII*		12
	*dfrA12*		17
	*dhfrXVII*		0
	*dhfrXII*		0
	*dhfrXIII + dfrA12*		10

a ABPC, ampicillin; CEZ, cefazolin; CTF, ceftiofur; DSM, dihydrostreptomycin; KM, kanamycin; GM, gentamicin; BCM, bicozamycin; OTC, oxytetracycline; CL, colistin; CP, chloramphenicol; NA, nalidixic acid; ERFX, enrofloxacin; TMP, trimethoprim.

b n, number of phenotypically resistant *E. coli* isolates used for PCR analysis and colony hybridization.

**Table 3:** AMR genes and plasmid incompatibility type

Incompatibility types^a^	Number of isolate detected	Resistance genes detected^b^	Sizes (kbp)	Number of different genotype of plasmid
FIB	39	*bla*_TEM_*, bla*_CTX-M_*, bla*_CMY_*, strA, strB, aphA1, aphAI-IAB, aacC2, tetA, tetB, tetC, catI, dhfrI, dhfrVII, dfrA12*	51–144	22
F	37	*bla*_TEM_*, bla*_CTX-M_*, bla*_CMY_*, strA, strB, aphA1, aphAI-IAB, aacC2, tetA, tetB, tetC, dhfrI, dhfrXIII, dfrA12*	46–144	24
FIA	16	*bla*_TEM_*, bla*_CTX-M_*, strA, strB, aphA1, aphAI-IAB, aacC2, tetB, catI, dhfrVII*	55–127	10
Y	16	*dfrA12*	67–68	5
N	14	*bla*_CTX-M_*, tetA, tetB, dhfrXIII, dfrA12*	106–109	4
A/C	8	*bla*_TEM_*, bla*_CMY_*, strA, strB, tetA, tetC, floR, dhfrI*	155–181	7
I1	5	*bla*_TEM_*, strA, strB, aphA1, aphAI-IAB*	51–53	3
P	1	Not detected	51	1
Others	45	*bla*_CTX-M_*, bla*_CMY_*, strA, strB, aphAI-IAB, tetA, tetC, floR, dfrA12*	2–774	45

a Incompatibility types of HI1, HI2, X, L/M, W, FIC, T, FIIAs, K and B/O were not detected.

b *bla*_TEM_, *bla*_CTX-M_ and *bla*_CMY_, beta-lactams resistance genes; *strA* and *strB*, DSM resistance gene; *aacC2*, GM resistance gene; *aphA1* and *aphAI-IAB*, KM resistance gene; *tetA*, *tetB* and *tetC*, OTC resistance gene; *catI* and *floR*, CP resistance gene; *dhfrI*, *dhfrVII*, *dhfrXIII* and *dfrA12*, TMP resistance gene.
