# Supplementary material for: Characterization of Multi-antibiotic-resistant Escherichia coli Isolated from Beef Cattle in Japan
Source: Microbes Environ. 2014 Apr 30;29(2):136–44. doi: 10.1264/jsme2.ME13173 (PMC4103519; doi:10.1264/jsme2.ME13173)
Supplement: Supplementary file 1 [file 29_136_s1.pdf]

TABLE S1. PCR primers

Target gene(s)	PCR primer sequence (5'-3')		Sizes of PCR products (bp)	Annealing temp (°C)	Reference[a]
	Forward	Reverse			
Antimicrobial resistance					
Beta-lactams (ABPC, CEZ, CTF)[b]					
bla_{TEM}	GAGTATTCAACATTTTCGT	ACCAATGCTTAATCAGTGA	857	50	6, 8, 9
bla_{SHV}	TCGCCTGTGTATTATCTCCC	CGCAGATAAATCACCACAATG	768	50	6, 8, 9
bla_{OXA}	TCAACTTTCAAGATCGCA	GTGTGTTTAGAATGGTGA	591	52	5, 9
$bla_{\text{CTX-M}}$	CGCTTTGCGATGTGCAG	ACCGCGATATCGTTGGT	550	52	5, 9
bla_{CMY}	GACAGCCTCTTTCTCCACA	TGGAACGAAGGCTACGTA	1000	50	5, 9
Aminoglycosides (DSM, GM, KM)					
strA[c]	CCTGGTGATAACGGCAATTC	CCAATCGCAGATAGAAGGC	546	55	8, 9
strB[c]	ATCGTCAAGGGATTGAAACC	GGATCGTAGAACATATTGGC	509	55	8, 9
aadB[d]	TCCAGAACCTTGACCGAAC	GCAAGACCTCAACCTTTTCC	700	50	6, 8, 9
aacC2[d]	CGGAAGGCAATAACGGAG	TCGAACAGGTAGCACTGAG	740	50	6, 8, 9
aac(3)- IV[d]	GTGTGCTGCTGGTCCACAGC	AGTTGACCCAGGGCTGTCGC	627	50	6, 8, 9
aphA1[e]	ATGGGCTCGCGATAATGTC	CTCACCGAGGCAGTTCCAT	600	50	6, 8, 9
aphA2[e]	GAACAAGATGGATTGCACGC	GCTCTTCAGCAATATCACGG	680	50	6, 8, 9
aadD[e]	ATATTGGATAAATATGGGGAT	TCCACCTTCCACTCACCGGTT	161	52	2, 9
aphA3[e]	CTGTTCCAAAGGTCCTGCACT	CAATTCGGCTAAGCGGCTGTC	unknown	52	2, 9
aphA/aph(3')-Id[e]	ATGGGCGCCTATCACAATTGG	TCGCCTCCAGCTCTTCGTAGA	unknown	52	2, 9
aphA7[e]	GGAAACACATAGATTGCTTTA	CTCCACATCTTGCCAAATCAT	unknown	52	2, 9
aphAI-IAB[e]	AAACGTCTTGCTCGAGGC	CAAACCGTTATTCATTCGTGA	unknown	52	2, 9
Kn[e]	ACTGGCTGCTATTGGGCGA	CGTCAAGAAGGCGATAGAAGG	515	52	2, 9
Kan[e]	GTGTTTATGGCTCTCTTGGTC	CCGTGTCGTTCTGTCCACTCC	unknown	52	2, 9
Tetracyclines					
tetA	GTGAAACCCAACATACCCC	GAAGGCAAGCAGGATGTAG	888	53	6, 8, 9
tetB	CCTTATCATGCCAGTCTTGC	ACTGCCGTTTTTTCGCC	774	53	6, 8, 9
tetC	ACTTGGAGCCACTATCGAC	CTACAATCCATGCCAACCC	881	53	6, 8, 9
tetD	AAACCATTACGGCATTCTGC	GACCGGATACACCATCCATC	787	53	5, 9
tetE	AAACCACATCCTCCATACGC	AAATAGGCCACAACCGTCAG	278	53	5, 9
tetG	GCTCGGTGGTATCTCTGCTC	AGCAACAGAATCGGGAACAC	468	53	5, 9
tetY	ACCGCACTCATTGTTGTC	TTCCAAGCAGCAACACAC	823	53	6, 9
tetS	CATAGACAAGCCGTTGACC	ATGTTTTTGGAACGCCAGAG	667	53	7, 9
Chloramphenicol					
cat I	AGTTGCTCAATGTACCTATAACC	TTGTAATTCATTAAGCATTCTGCC	547	50	6, 8, 9
catII	ACACTTTGCCCTTTATCGTC	TGAAAGCCATCACATACTGC	543	50	6, 9
catIII	TTCGCCGTGAGCATTTTG	TCGGATGAGTATGGGCAAC	286	50	6, 9
floR	CGCCGTCATTCCTCACCTTC	GATCACGGGCCACGCTGTGTC	215	50	6, 8, 9
cmlA	TTGCAACAGTACGTGACAT	ACACAACGTGTACAACCAG	293	55	8, 9
Trimethoprim					
dhfrI	AAGAATGGAGTTATCGGGAATG	GGGTAAAAACTGGCCTAAAATTG	391	50	6, 8, 9
dhfr V	CTGCAAAAGCGAAAAACGG	AGCAATAGTTAATGTTTGAGCTAAAG	432	50	6, 8, 9
dhfr VII	GGTAATGGCCCTGATATCCC	TGTAGATTTGACCGCCACC	265	50	6, 8, 9
dhfr IX	TCTAAACATGATTGTCGCTGTC	TTGTTTTCAGTAATGGTCGGG	462	50	6, 8, 9
dhfrXIII	CAGGTGAGCAGAAGATTTTT	CCTCAAAGGTTTGATGTACC	294	50	6, 8, 9
dfrA12	ACTCGGAATCAGTACGCA	GTGTACGGAATTACAGCT	462	53	3, 9
dhfrXVII	GTCGCCCTAAAACAAAGTTA	TGTAAACTGAGATACCCGC	195	53	4, 9
dhfrXII	AAATTCCGGGTGAGCAGAAG	GATTGGTAAGGCAGTTGCCC	429	53	4, 9
Incompatibility type					
HI1	GGAGCGATGGATTACTTCAGTAC	TGCCGTTTCACCTCGTGAGTA	471	55	1
HI2	TTTCTCCTGAGTCACCTGTTAACAC	GGCTCACTACCGTTGTCATCCT	644	55	1
I1	CGAAAGCCGGACGGCAGAA	TCGTCGTTCCGCCAAGTTCGT	139	55	1
X	AACCTTAGAGGCTATTTAAGTTGCTGAT	TGAGAGTCAATTTTTATCTCATGTTTTAGC	376	55	1
L/M	GGATGAAAACTATCAGCATCTGAAG	CTGCAGGGGCGATTCTTTAGG	785	55	1
N	GTCTAACGAGCTTACCGAAG	GTTTCAACTCTGCCAAGTTC	559	55	1
FIA	CCATGCTGGTTCTAGAGAAGGTG	GTATATCCTTACTGGCTTCCGCAG	462	55	1
FIB	GGAGTTCTGACACACGATTTTCTG	CTCCCGTCGCTTCAGGGCATT	702	55	1
W	CCTAAGAACAACAAAGCCCCCG	GGTGCGCGGCATAGAACCGT	242	55	1
Y	AATTCAAACAACACTGTGCAGCCTG	GCGAGAATGGACGATTACAAAACTTT	765	55	1
P	CTATGGCCCTGCAAACGCGCCAGAAA	TCACGCGCCAGGGCGCAGCC	534	55	1
FIC	GTGAACTGGCAGATGAGGAAGG	TTCTCCTCGTCGCCAAACTAGAT	262	55	1
A/C	GAGAACCAAAGACAAAGACCTGGA	ACGACAAACCTGAATTGCCTCCTT	465	55	1
T	TTGGCCTGTTTGTGCCTAAACCAT	CGTTGATTACACTTAGCTTTGGAC	750	55	1
FIIAs	CTGTCGTAAGCTGATGGC	CTCTGCCACAAACTTCAGC	270	55	1
F	TGATCGTTTAAGGAATTTTG	GAAGATCAGTCACACCATCC	270	52	1
K	GCGGTCCGGAAAGCCAGAAAAC	TCTTTCACGAGCCCGCCAAA	160	55	1
B/O	GCGGTCCGGAAAGCCAGAAAAC	TCTGCGTTCCGCCAAGTTCGA	159	55	1

[a] Reference; 1, Carattoli A., et al. 2005. J. Microbiol. Methods 63:219-228; 2, Frana T. S., et al. 2001. Appl. Environ. Microbiol. 67:445-448; 3, Guerra B., et al. 2001. Antimicrob. Agents Chemother. 45:1305-1308; 4, Harada K., et al. 2006. Am. J. Vet. Res. 67:230-235; 5, Ishida Y., et al. 2010. J. Vet. Med. Sci. 72:727-734; 6, Maynard C., et al. 2004. J. Clin. Microbiol. 42:5444-5452; 7, Ng L.-K., et al. 1999. Antimicrob. Agents Chemother. 43:3018-3021; 8, Rosengren L. B., et al. 2009. Appl. Environ. Microbiol. 75:1373-1380; 9, Yamamoto S., et al. 2013. J. Food Prot. 76:394-404

[b] ABPC, ampicillin; CEZ, cefazolin; CTF, ceftiofur.

[c] Dihydrostreptomycin (DSM) resistance gene.

[d] Gentamicin (GM) resistance gene.

[e] Kanamycin (KM) resistance gene.

Table S2. Characterization of plasmids, AMR genes, and incompatibility types

Group No.	Plasmid	Resistance genes[a]	Size (kbp)	Incompatibility types
1	pGC1-2-4-A	bla_{CMY}, *strA, strB, tetA, tetC, floR*	774	
2	pGC1-2-4-B	bla_{CMY}, *strA, strB, tetA, tetC, floR*	442	
3	pQD1-3-ER-4-A	*strA, strB*	342	
4	pQD1-3-ER-4-B	*tetC*	200	
5	pGC1-3-GR-4-A	bla_{TEM}, bla_{CMY}, *strA, strB, tetA, tetC, floR*	181	A/C
6	pQD1-3-ER-4-C	*strA, strB, tetA, tetC*	181	
7	pGC1-2-GR-1-A	bla_{TEM}, bla_{CMY}, *strA, strB, tetA, tetC, floR*	178	A/C
8	pGC1-1-ER-2-A, pGC1-2-4-C	bla_{TEM}, bla_{CMY}, *strA, strB, tetA, tetC, floR*	176	A/C
9	pGC1-2-GR-2-A	bla_{TEM}, *strA, strB, tetA, tetC, floR, dhfrI*	172	A/C
10	pGC1-2-GR-3-A	bla_{TEM}, *strA, strB, tetA, tetC, floR, dhfrI*	168	A/C
11	pQD1-3-ER-2-E, pQD1-5-GR-1-E	Nothing	168	
12	pGC1-2-GR-5-A	bla_{TEM}, *strA, strB, tetA, tetC, floR, dhfrI*	159	A/C
13	pQD1-1-ER-1-H, pQD1-1-ER-3-E, pQD1-1-ER-5-E, pQD1-3-FR-1-E, pQD1-3-FR-2-E, pQD1-3-FR-3-E, pQD1-3-FR-4-E, pQD1-3-FR-5-E	Not detected	159	
14	pGC1-3-GR-2-A	bla_{TEM}, *strA, strB, tetA, tetC, floR, dhfrI*	155	A/C
15	pQD1-3-2-E, pQD1-5-9-E, pQD1-1-ER-2-E, pQD1-1-ER-4-E	Nothing	155	
16	pGC1-3-GR-4-B	bla_{TEM}, bla_{CMY}, *strA, strB, tetC*	144	F, FIB
17	pGC1-1-ER-2-B	bla_{TEM}, bla_{CMY}, *strA, strB, tetA*	140	F, FIB
18	pGC1-2-4-D	bla_{TEM}, bla_{CMY}, *strA, strB, tetC*	140	F, FIB
19	pGC1-2-GR-1-B	bla_{TEM}, *strA, strB*	140	F, FIB
20	pGC1-2-GR-2-B	bla_{TEM}, *strA, strB, dhfrI*	140	F, FIB
21	pGC1-2-GR-3-B	bla_{TEM}, *strA, strB, tetC, dhfrI*	133	F, FIB
22	pGC1-2-GR-5-B	bla_{TEM}, *strA, strB, dhfrI*	129	F, FIB
23	pQB1-2-GR-3-A, pQB1-2-GR-4-A	*strA, strB, aphAI-IAB, tetB*	127	F, FIA, FIB
24	pQB1-3-GR-2-A	*strA, strB, aphA1, aphAI-IAB, tetB*	123	F, FIA, FIB
25	pGC1-3-GR-2-B	bla_{TEM}, *strA, strB, dhfrI*	123	F, FIB
26	pQB1-1-GR-1-A, pQB1-2-GR-5-A, pQB1-3-GR-3-A	*strA, strB, aphAI-IAB, tetB*	120	F, FIA, FIB
27	pQB1-4-GR-3-A, pQB1-4-GR-5-A, pQB1-5-GR-1-A	*strA, strB, aphAI-IAB, tetB*	120	F, FIB
28	pQB1-3-GR-4-A	*strA, strB, aphAI-IAB, tetB*	114	F, FIA, FIB
29	pQB1-4-GR-1-A, pQB1-4-GR-2-A	*strA, strB, aphAI-IAB, tetB*	111	F, FIB
30	pGC1-2-4-E	bla_{CMY}	109	
31	pQB1-3-GR-5-A	*strA, strB, aphAI-IAB, tetB*	109	F, FIA, FIB
32	pQD1-3-2-A	$bla_{\text{CTX-M}}$, *tetA, tetB, dfrA12*	109	F, N
33	pQD1-5-9-A, pQD1-1-ER-4-A, pQD1-1-ER-5-A, pQD1-3-FR-1-A, pQD1-5-GR-1-A	$bla_{\text{CTX-M}}$, *tetA, tetB, dhfrXIII, dfrA12*	109	F, N
34	pQD1-1-ER-1-A, pQD1-1-ER-2-A, pQD1-1-ER-3-A	$bla_{\text{CTX-M}}$, *tetA, tetB, dfrA12*	106	F, N
35	pQD1-3-FR-2-A, pQD1-3-FR-3-A, pQD1-3-FR-4-A, pQD1-3-FR-5-A, pQD1-3-ER-2-A	$bla_{\text{CTX-M}}$, *tetA, tetB, dhfrXIII, dfrA12*	106	F, N
36	pKT1-2-ER-3-A	*strA, aphAI-IAB, tetA*	103	F
37	pSA1-6-GR-1-A, pSA1-12-ER-1-A	bla_{TEM}, *strA, strB, aphA1, aphAI-IAB, aacC2, tetB, catI, dhfrVII*	96	FIA, FIB
38	pSA1-12-GR-2-A, pSA1-12-GR-4-A, pSA1-12-GR-5-A	bla_{TEM}, *strA, strB, aphA1, aphAI-IAB, aacC2, tetB, catI, dhfrVII*	96	FIA
39	pSA1-12-GR-3-A	bla_{TEM}, *strA, aphA1, aphAI-IAB, aacC2, tetB, catI, dhfrVII*	96	FIA
40	pSA1-12-GR-1-A	bla_{TEM}, *strA, strB, aphA1, aphAI-IAB, aacC2, tetB, catI, dhfrVII*	93	FIA, FIB
41	pKT1-2-ER-3-B	*tetA*	89	
42	pSA1-4-1-A	Not detected	80	
43	pQD1-3-ER-4-D	Not detected	78	Y
44	pQB1-2-GR-5-C	Not detected	78	
45	pQB1-5-GR-1-C	Not detected	76	
46	pQD1-1-ER-1-B	$bla_{\text{CTX-M}}$	70	
47	pQD1-3-2-B, pQD1-5-9-B, pQD1-1-ER-2-B, pQD1-1-ER-3-B, pQD1-1-ER-4-B, pQD1-1-ER-5-B, pQD1-3-FR-1-B, pQD1-5-GR-1-B	*dfrA12*	69	Y
48	pSA1-6-GR-1-B	Not detected	69	Y
49	pQD1-3-FR-2-B, pQD1-3-FR-3-B, pQD1-3-FR-4-B, pQD1-3-FR-5-B, pQD1-3-ER-2-B	*dfrA12*	67	Y
50	pGC1-2-4-F	bla_{CMY}, *tetA*	64	
51	pQD1-1-ER-1-C	$bla_{\text{CTX-M}}$, *strA, strB, aacC2*	55	FIA, FIB
52	pQD1-5-9-C, pQD1-1-ER-2-C, pQD1-1-ER-3-C, pQD1-1-ER-4-C, pQD1-1-ER-5-C	*strA, strB, aacC2, dfrA12*	55	FIB
53	pQB1-2-GR-3-B, pQB1-2-GR-4-B	bla_{TEM}, *strA, strB, aphA1, aphAI-IAB*	53	F, I1
54	pQD1-3-2-C, pQD1-3-FR-1-C, pQD1-3-FR-3-C, pQD1-5-GR-1-C	*strA, strB, aacC2, dfrA12*	53	FIB
55	pQB1-3-GR-5-B	*strB*	52	I1
56	pQD1-3-FR-2-C, pQD1-3-FR-4-C, pQD1-3-FR-5-C, pQD1-3-ER-2-C	*strA, strB, aacC2, dfrA12*	52	FIB
57	pKT1-2-ER-3-C	Not detected	51	FIB, P
58	pQB1-4-GR-2-B	Not detected	51	I1
59	pSA1-4-1-B	Not detected	51	
60	pQB1-2-GR-5-B	bla_{TEM}, *strA, aphA1, aphAI-IAB*	49	F
61	pGC1-2-4-G	*tetA*	47	
62	pQB1-3-GR-3-B	bla_{TEM}, *strA, aphA1, aphAI-IAB*	46	F
63	pQB1-5-GR-1-B	bla_{TEM}, *aphA1, aacC2*	46	F
64	pQD1-1-ER-1-D	$bla_{\text{CTX-M}}$	45	
65	pQD1-3-2-D, pQD1-5-9-D, pQD1-1-ER-2-D, pQD1-1-ER-3-D, pQD1-1-ER-4-D, pQD1-1-ER-5-D, pQD1-3-FR-1-D, pQD1-3-FR-2-D, pQD1-3-FR-3-D, pQD1-3-FR-5-D, pQD1-3-ER-2-D, pQD1-5-GR-1-D	*dfrA12*	42	
66	pSA1-6-GR-1-C	Not detected	42	
67	pQD1-3-FR-4-D	*dfrA12*	41	
68	pQB1-2-GR-3-C, pQB1-2-GR-4-C	*strA, aphAI-IAB*	39	
69	pKT1-2-ER-3-C	*tetC*	35	
70	pSA1-12-ER-1-B	*strA*	30	
71	pQD1-1-ER-1-I	Not detected	18	
72	pQD1-3-ER-4-F	*tetC*	14	
73	pQD1-1-ER-1-F	*aphAI-IAB, floR*	13	
74	pQD1-1-ER-5-F, pQD1-3-FR-5-F	Not detected	13	
75	pQD1-1-ER-1-E	$bla_{\text{CTX-M}}$	12	
76	pQD1-1-ER-2-F	Not detected	12	
77	pQD1-3-ER-4-G	*strA, strB, aphAI-IAB, tetA, tetC, floR*	11	
78	pQD1-1-ER-5-G, pQD1-1-ER-2-G, pQD1-3-FR-3-F, pQD1-3-FR-5-G	Not detected	11	
79	pQD1-3-ER-4-E	*strA, strB, tetC*	10	
80	pQD1-5-9-F, pQD1-1-ER-3-F, pQD1-3-FR-2-F	Not detected	10	
81	pQD1-3-FR-1-F, pQD1-3-FR-4-F, pQD1-3-ER-2-F, pQD1-5-GR-1-F, pQD1-3-2-F	Not detected	9	
82	pQD1-3-ER-4-H	*strA, strB, aphAI-IAB, tetA*	8	
83	pQD1-5-9-G, pQD1-1-ER-2-H, pQD1-1-ER-3-G, pQD1-1-ER-4-F, pQD1-3-FR-1-G, pQD1-3-FR-2-G, pQD1-1-ER-5-H, pQD1-3-FR-4-G, pGC1-1-ER-2-E, pQD1-1-ER-1-J, pQB1-3-GR-4-B, pQD1-3-ER-2-G, pQD1-5-GR-1-G	Not detected	8	
84	pGC1-1-ER-2-C	*aphAI-IAB*	6	
85	pQD1-1-ER-1-G	*aphAI-IAB, floR*	6	
86	pQB1-4-GR-5-B, pQB1-5-GR-1-D, pGC1-2-GR-1-D, pGC1-3-GR-2-C, pQB1-4-GR-3-B, pGC1-1-ER-2-F, pQB1-3-GR-2-C, pQD1-3-ER-4-J, pGC1-2-GR-2-C, pGC1-2-GR-3-C, pGC1-2-GR-5-C	Not detected	5	
87	pGC1-1-ER-2-D, pGC1-2-GR-1-C, pGC1-3-GR-4-C, pQD1-3-ER-4-I	*aphAI-IAB*	4	
88	pQB1-3-GR-2-B	*aphAI-IAB, floR*	4	
89	pGC1-3-GR-2-D, pQB1-2-GR-3-D, pQB1-3-GR-5-C, pQB1-1-GR-1-B, pQB1-2-GR-4-D, pQB1-2-GR-5-D, pQB1-3-GR-3-C, pQB1-3-GR-4-C, pQB1-4-GR-1-B, pQB1-4-GR-2-C, pGC1-2-4-H, pGC1-2-GR-5-D, pGC1-2-GR-2-D, pGC1-2-GR-3-D	Not detected	4	
90	pSA1-4-1-C, pSA1-12-GR-4-B, pSA1-12-GR-5-B	Not detected	3	
91	pSA1-12-GR-1-B	*aphAI-IAB*	2	
92	pSA1-12-GR-2-B, pSA1-6-GR-1-D, pSA1-12-GR-3-B, pSA1-12-ER-1-C	Not detected	2	

[a] bla_{TEM}, $bla_{\text{CTX-M}}$ and bla_{CMY}, beta-lactams resistance genes; *strA* and *strB*, DSM resistance gene; *aacC2*, GM resistance gene; *aphA1* and *aphAI-IAB*, KM resistance gene; *tetA*, *tetB* and *tetC*, OTC resistance gene; *catI* and *floR*, CP resistance gene; *dhfrI*, *dhfrVII*, *dhfrXIII* and *dfrA12*, TMP resistance gene.
